# Implementation of Field-Programmable Gate Array Platform for Object Classification Tasks Using Spike-Based Backpropagated Deep Convolutional Spiking Neural Networks

**DOI:** 10.3390/mi14071353

**Published:** 2023-06-30

**Authors:** Vijay Kakani, Xingyou Li, Xuenan Cui, Heetak Kim, Byung-Soo Kim, Hakil Kim

**Affiliations:** 1 Integrated System Engineering, Inha University, 100 Inharo, Nam-gu, Incheon 22212, Republic of Korea; vjkakani@inha.ac.kr; 2 Electrical and Computer Engineering, Inha University, 100 Inharo, Nam-gu, Incheon 22212, Republic of Korea; 22202326@inha.edu; 3 Information and Communication Engineering, Inha University, 100 Inharo, Nam-gu, Incheon 22212, Republic of Korea; xncui@inha.ac.kr; 4 Research and Development, Korea Electronics Technology Institute, 25 KETI, Saenari-ro, Seongnam-si 13509, Republic of Korea; htkim@keti.re.kr (H.K.); bskim4k@keti.re.kr (B.-S.K.)

**Keywords:** field-programmable gate arrays, neuromorphic image processing, object classification performance, spiking neural networks

## Abstract

This paper investigates the performance of deep convolutional spiking neural networks (DCSNNs) trained using spike-based backpropagation techniques. Specifically, the study examined temporal spike sequence learning via backpropagation (TSSL-BP) and surrogate gradient descent via backpropagation (SGD-BP) as effective techniques for training DCSNNs on the field programmable gate array (FPGA) platform for object classification tasks. The primary objective of this experimental study was twofold: (i) to determine the most effective backpropagation technique, TSSL-BP or SGD-BP, for deeper spiking neural networks (SNNs) with convolution filters across various datasets; and (ii) to assess the feasibility of deploying DCSNNs trained using backpropagation techniques on low-power FPGA for inference, considering potential configuration adjustments and power requirements. The aforementioned objectives will assist in informing researchers and companies in this field regarding the limitations and unique perspectives of deploying DCSNNs on low-power FPGA devices. The study contributions have three main aspects: (i) the design of a low-power FPGA board featuring a deployable DCSNN chip suitable for object classification tasks; (ii) the inference of TSSL-BP and SGD-BP models with novel network architectures on the FPGA board for object classification tasks; and (iii) a comparative evaluation of the selected spike-based backpropagation techniques and the object classification performance of DCSNNs across multiple metrics using both public (MNIST, CIFAR10, KITTI) and private (INHA_ADAS, INHA_KLP) datasets.

## 1. Introduction

Innovations in artificial neural networks [1,2] and vision-based technologies [3,4,5] have spawned intelligent applications in a variety of fields [6,7,8,9,10,11], despite some limitations in low-cost computing [12]. Due to developments in spiking neural networks (SNNs), neuromorphic processing units inspired by the brain have gained popularity [13]. In SNNs, neurons communicate with one another by means of spike patterns, transmitting spike information from the input neuron to other interconnected neurons, and ultimately to the output neuron [14]. SNNs have been utilized as the neuromorphic processing units for artificial intelligence tasks requiring efficient energy consumption [15]. SNNs are considered the third iteration of artificial neural networks (ANNs) and possess nearly the same computational capability as ANNs [16]. SNNs are distinguished from ANNs predominantly by their discrete output spikes. In contrast to ANNs, which have continuous values as neuron responses, SNNs have discrete pulses that are typically repetitive due to membrane potential [17,18,19,20]. The operation of SNNs is defined by the leaky integrate fire neuron (LIF), which is dependent on the membrane potential dynamics of the neuron. Presently, neuromorphic processors such as IBM’s TrueNorth and Intel’s Loihi enable researchers to deploy spiking neural networks with a performance that is comparable to that of PC-based convolutional neural networks [21,22]. The aforementioned neuromorphic hardware devices are costly in terms of initial price and maintenance [23]. This has encouraged numerous researchers to implement spiking neural networks on inexpensive field-programmable gate array (FPGA) boards [24,25,26,27,28].

### 1.1. Motivation

To operate object classification and detection algorithms, a number of mid-sized businesses favor low-power chips, particularly in the autonomous vehicle industry. Based on previous interactions with these companies in the current demographics, their primary need is to acquire a low-cost, low-power computing bench with considerable accuracy and processing speed for their autonomous systems. Typically, the final autonomous system design for these institutions includes redundant benchmarking protocols to evaluate the precision and processing speed provided by a low-power computing bench. In the future, these mid-sized companies might use neural networks for power analysis [29] and electrical load prediction [30,31] to compare low-power FPGAs to other computer platforms. Therefore, there is a need for reporting the shortcomings and borderline advantages of deploying powerful DCSNNs to attain higher accuracy on low-power FPGAs at the expense of processing latency.

### 1.2. Purpose of Study

**Figure 1:**
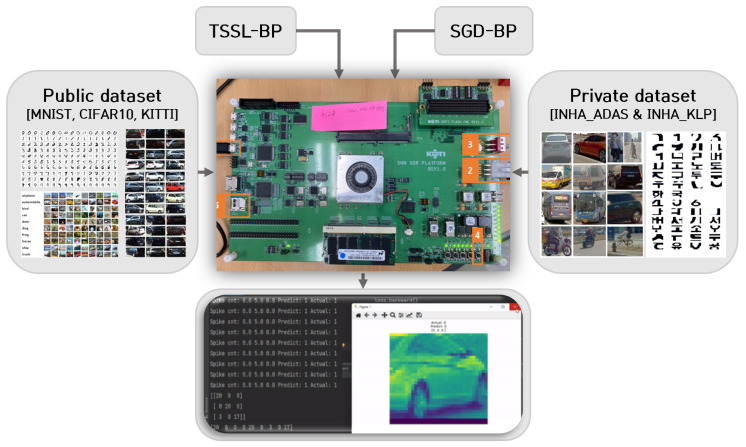
Implementation schematic of DCSNN using spike-based backpropagation on FPGA platform.

The unique objective of this study was to report and assist mid-level autonomous vehicle manufacturers with the potential deployment of low-power networks such as DCSNNs for deep learning tasks on low-power FPGA boards. In addition, hybrid networks such as DCSNNs could match the accuracy of their ANN counterparts with regard to object classification tasks on a variety of public and private datasets. The research conducted in this study regarding the deployment of DCSNNs on low-cost FPGA boards and the accuracy and processing time latency with respect to MNIST, CIFAR10, KITTI, INHA_ADAS, and INHA_KLP could inform researchers and businesses in this field about the limitations and distinctive perspectives of this approach. The overall study analysis is depicted in Figure 1 below.

## 2. Literature Review

Multiple studies have been conducted to attain a comparable level of efficiency for SNNs using backpropagation techniques [32,33,34,35,36,37,38,39,40]. Some have utilized the neuron action potential timing information to infer and distinguish the timing information for potential backpropagation [34,41]. The limitation of these methodologies is that a reduction in the neuronal firing rates eventually leads to a decline in the network’s capacity. To circumvent this issue, unsupervised learning techniques such as spike-time-dependent plasticity (STDP) have been utilized to train SNNs. The STDP mechanism was utilized to design reward-modulated STDP for supervised learning, enabling networks to perform object recognition and autonomous tasks, in [42,43]. In a manner of speaking, these mechanisms have a high energy requirement and will degrade the overall efficacy of the system when implemented on deeper SNNs [44,45]. Several of these studies employed spiking non-linearity to approximate the discontinuous spiking activation function, thereby flattening the activation function and rendering the SNN continuously differentiable [46]. Recent studies employing techniques such as surrogate gradient descent (SGD) [46,47,48] and temporal spike sequence learning (TSSL) [49] have substantially improved the consistency of the training process for deeper SNNs while maintaining the accuracy of the SNNs at the same level as that of ANNs on standard PC hardware.

These authors conducted a few exploratory analyses on SGD-BP SNNs executed on PCs in conjunction with NVIDIA TX2 embedded platforms [47]. Similarly, the authors developed a number of deeper SNN architectures trained with SGD-BP and implemented on an embedded board [48,50]. In addition, a literature review of a few potential hybrid networks (involving SNNs with convolutions) on the FPGA platform was conducted to assess the scope of this study; the pertinent information is presented in Table 1. The networks compared in the literature review table alongside the proposed study are all either SNNs or hybrid networks (convolutional SNNs). However, no known study except for [49] has utilized deeper convolutional layers coupled with either integrated fire (IF) or leaky integrate fire (LIF) SNN layers. This combination is very powerful, as it harnesses the power of a convolutional filter alongside the spiking mechanism of IF or LIF neurons. When using low-power FPGA boards, as in several studies [49,51,52,53,54,55,56], it is challenging to balance both the deeper convolutions and spiking mechanisms. The current study was able to overcome several of the challenges faced by other works because of the following:

- We hosted deeper convolutions alongside SNNs with very few parameters compared to [49] and were still able to achieve similar accuracy over the MNIST and CIFAR10 datasets.
- We employed both real-valued and Poisson distribution spikes as input encoding schemes to capture most of the information before processing them through DCSNNs, which were not used in [49,51,52,53,54,55,56].
- We tested the DCSNNs on automotive relevant datasets such as KITTI, INHA_ADAS, and INHA_KLP as opposed to just MNIST and CIFAR10, as was the case in [49,52,54,55,56].
- We customized the proposed SGD-BP to fit the low-power needs of several target medium-sized intelligent vehicle industries in the form of FPGA implementation while preserving accuracy.

## 3. Spiking Schematic Design Framework

### 3.1. Spiking Neuron Model

The spiking neural network employed in this study was constructed by the adaptation of the LIF neuron model [13]. According to the LIF neuron model, the input spike train flows from the presynaptic neuron *v* to the postsynaptic neuron *u*. The input spike train can be denoted by $X_{v}\left(t\right)=\sum_{f_{v}^{\left(t\right)}}\delta \left(f-f_{v}^{\left(t\right)}\right)$, where $f_{v}^{\left(t\right)}$ represents the firing time of the presynaptic neuron *v*. The postsynaptic current $J_{v}\left(t\right)$ is produced from the incoming spikes through the synaptic connection between neuron *v* and neuron *u*. The membrane potential voltage $P_{u}\left(t\right)$ for the postsynaptic neuron *u* at a given time *t* is represented by

(1) $$\tau_{p}\frac{dP_{u}\left(t\right)}{dt}=-P_{u}\left(t\right)+R_{0}\sum_{v}Q_{uv}J_{v}\left(t\right)+r_{u}\left(t\right),$$

where $R_{o}$ is the leaky resistance of the LIF neuron, $\tau_{p}$ is the membrane potential time constant, $Q_{uv}$ is the weight of the synaptic connection between the presynaptic and postsynaptic neurons, $J_{v}\left(t\right)$ is the postsynaptic current inculcated by the presynaptic neuron spike, and $r_{u}\left(t\right)$ is the reset mechanism in the spiking activity. The postsynaptic current and the reset mechanism can be denoted as

(2) $$J_{v}\left(t\right)=\left(\alpha *X_{v}\right)\left(t\right),r_{u}\left(t\right)=\left(\beta *X_{u}\right)\left(t\right),$$

where α(·) and β(·) are the response mechanism kernel and reset mechanism kernel, respectively. Accordingly, the first-order spike response is denoted in conjunction with a synaptic time constant $\tau_{s}$ as

(3) $$\tau_{s}\frac{J_{v}\left(t\right)}{dt}=-J_{v}\left(t\right)+X_{v}\left(t\right).$$

The membrane potential is reduced by the reset mechanism for each neuron firing period by a specific firing equilibrium value. By applying the Euler method to 1, the membrane potential is simplified as

(4) $$P_{u}\left[t\right]=\left(1-\frac{1}{\tau_{p}}\right)P_{u}\left[t-1\right]+\sum_{v}Q_{uv}J_{v}\left[t\right]+r_{u}\left[t\right].$$

The overall firing mechanism is then followed by the reset scheme to obtain the output of the firing neuron as

(5) $$X_{v}\left[t\right]=H\left(P_{u}\left(t\right)-V_{Eq}\right),$$

where $V_{Eq}$ is the firing equilibrium or threshold, and H(·) is the step function.

### 3.2. Deep Convolutional Spiking Neural Networks (DCSNNs)

**Figure 2:**
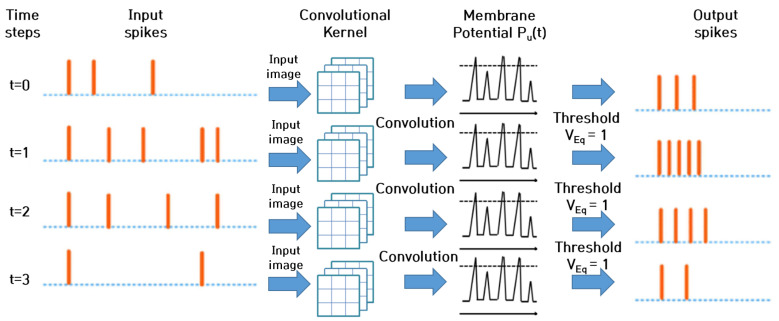
Schematic of deep convolutional spiking neural networks.

The combination of convolutional kernels and pooling layers with spiking neural network components results in DCSNNs. This study employed such architectures with additional layers to perform classification tasks. As input spikes pass through various layers, the training process occurs. When the input spike train is processed by the filters in the convolutional layers, the input current is estimated. At each time step, the input current determines the membrane potential $P_{u}\left(t\right)$ of the neuron. When the neuron’s $P_{u}\left(t\right)$ exceeds the threshold value $V_{Eq}$, both the neuron’s spikes and the membrane potential revert to their initial values of zero. In contrast, the value of $P_{u}\left(t\right)$ is regarded as residual leakage over the course of the subsequent time steps. The pooling layers in the DCSNN function are similar to those in ANNs; however, the spike representation of the input image corresponding to spatial information is governed by either average [53,58] or maximum [59] pooling. A schematic of DCSNNs is depicted in Figure 2. These factors contribute to the stability of the training of deep convolutional spiking neural networks.

## 4. Training DCSNNs with Backpropagation

### 4.1. TSSL-BP for DCSNNs

The temporal spike loss function proposed in [49] was formulated as the sum of the squared error with respect to each time step for all neurons. This enabled the calculation of the difference between the desired spikes $D_{sp}=[D_{sp}{|}_{t=t_{0}},D_{sp}{|}_{t=t_{1}},\ldots ,D_{sp}{|}_{t=t_{N_{t}}}]$ and produced (actual) spikes $S_{sp}=[S_{sp}{|}_{t=t_{0}},S_{sp}{|}_{t=t_{1}},\ldots ,S_{sp}{|}_{t=t_{N_{t}}}]$, where $D_{sp}{|}_{t}$ and $S_{sp}{|}_{t}$ are the desired and produced (actual) firing events, respectively, at time *t* for the output neurons, with the number of total time steps being $N_{t}$. The temporal spike loss function was calculated as

(6) $$\begin{array}{cc} \; & L_{temp.sp}=\sum_{n=0}^{N_{t}}\xi_{TSSL}\left[t_{n}\right];\\ & \xi_{TSSL}\left[t_{n}\right]=\sum_{n=0}^{N_{t}}\frac{1}{2}{\left(\left(\Delta *D_{\mathrm{sp}}\right)\left[t_{n}\right]-\left(\Delta *S_{\mathrm{sp}}\right)\left[t_{n}\right]\right)}^{2}, \end{array}$$

where $\xi_{TSSL}\left[t\right]$ represents the error at time *t*, and ▵(·) represents a function that yields the Van Rossum difference between $D_{sp}{|}_{t}$ and $S_{sp}{|}_{t}$.

### 4.2. SGD-BP for DCSNNs

The loss in the surrogate gradient descent is defined with respect to the integral over the time steps, where the difference in desired spikes $D_{sp}=[D_{sp}{|}_{t=t_{0}},D_{sp}{|}_{t=t_{1}},\ldots ,D_{sp}{|}_{t=t_{N_{t}}}]$ and actual spikes $S_{sp}=[S_{sp}{|}_{t=t_{0}},S_{sp}{|}_{t=t_{1}},\ldots ,S_{sp}{|}_{t=t_{N_{t}}}]$ are coupled by the amount of membrane potential $P(t_{n})$ [46,60]:

(7) $$\xi_{SGD}\left[t\right]=\int_{0}^{N_{t}}\left[S_{sp}\left(t_{n}\right)-D_{sp}\left(t_{n}\right)\right]*P\left(t_{n}\right)*dt_{n},$$

where $\xi_{SGD}\left[t\right]$ represents the SGD error at time *t* between $D_{sp}{|}_{t}$ and $S_{sp}{|}_{t}$. The membrane potential $P(t_{n})$ influences the loss function in the case of SGD, as the growth in membrane potential corresponds to the reduction in loss when the output spike is absent $[S_{sp}\left(t_{n}\right)-D_{sp}\left(t_{n}\right)]<0$, and vice versa when $[S_{sp}\left(t_{n}\right)-D_{sp}\left(t_{n}\right)]>0$. Thus, the Van Rossum distance is used to achieve the stable control of the loss function. Additionally, the DCSNN uses convolutional kernels, which was taken into consideration according to previous studies [38] to further optimize the loss function. The entire loss function with respect to the total time period $N_{t}$ was calculated in the presence of convolutional kernel a(t)=1 as

(8) $$\xi_{SGD}\left[t\right]=\int_{0}^{N_{t}}\left[\left(a*S_{sp}\right)\left(t_{n}\right)-\left(a*D_{sp}\right)\left(t_{n}\right)\right]\left(a*P\right)\left(t_{n}\right)dt_{n}.$$

Considering the Heaviside step function *H*, and with $Z_{gt}\to \left[0,1\right]$ being the ground-truth labels for classification and $Z=\int_{0}^{N_{t}}S_{sp}\left(t_{n}\right)dt_{n}$ being the actual output of the network, the loss function could be modified to

(9) $$\xi_{SGD}\left[t\right]=H\left[Z\left(t_{n}\right)-Z_{gt}\left(t_{n}\right)\right]\int_{0}^{N_{t}}P\left(t_{n}\right)dt_{n}$$

The membrane potential and errors associated with the different loss expressions presented in (7)–(9) could be combined using the background presented in [46] as follows:

(10) $$\begin{array}{cc} \; & \xi_{SGD}\left[t\right]=\frac{1}{2}\int_{0}^{N_{t}}{\left[S_{sp}\left(t_{n}\right)-D_{sp}\left(t_{n}\right)\right]}^{2}dt_{n};\\ \; & \xi_{SGD}\left[t\right]=\frac{1}{2}{\left(H\left[Z\left(t_{n}\right)-Z_{gt}\left(t_{n}\right)\right]\right)}^{2} \end{array}$$

The effect of surrogate gradient with respect to membrane potential on the loss function was derived in [46,60] such that the differentiable output of the actual spikes was directly associated with the function of the membrane potential coupled with a change in the membrane potential as follows:

(11) $$\frac{d[S_{sp}\left(t_{n}\right)]}{dt_{n}}\to f\left(P\left(t_{n}\right)\right)*\frac{d[P\left(t_{n}\right)]}{dt_{n}}.$$

The function of the membrane potential was the combination of the hyperparameter χ, gradient thickness *c*, and difference between the membrane potential and equilibrium threshold $f\left(P\left(t_{n}\right)\right)=\frac{\chi }{{\left[1+c\left(P\left(t_{n}\right)-V_{Eq}\right)\right]}^{2}}$; thus, (11) could be written as

(12) $$\frac{d[S_{sp}\left(t_{n}\right)]}{dt_{n}}\to \frac{\chi }{{\left[1+c\left(P\left(t_{n}\right)-V_{Eq}\right)\right]}^{2}}*\frac{d[P\left(t_{n}\right)]}{dt_{n}}.$$

## 5. FPGA Schematic and Network Architecture

### 5.1. FPGA Design and Data Processing

**Figure 3:**
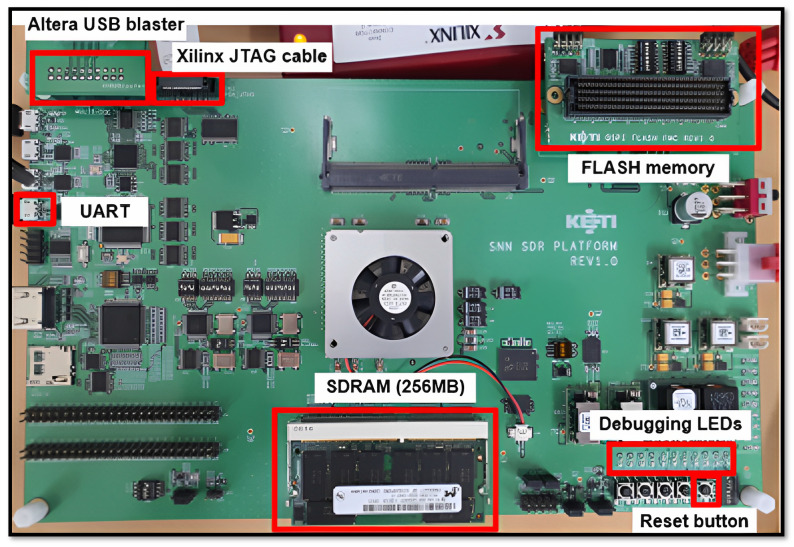
The external view of the on-chip FPGA spiking neural network processor.

**Figure 4:**
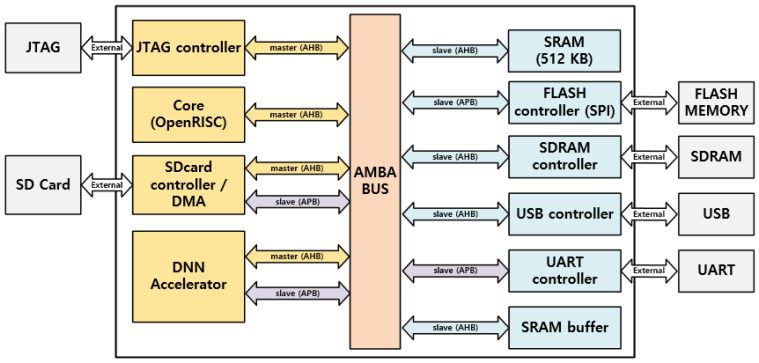
The overall block schematic of the FPGA board design.

The external view of the SNN processor on-chip FPGA board used in this investigation, which was intended to host spiking neural networks, is depicted in Figure 3. External components such as JTAG, an SD card, flash memory, USB, UART, and SDRAM made up the FPGA board. Consequently, the internal components consisted of a JTAG controller; OpenRISC core; SDcard controller; DNN accelerator; 512 KB of SRAM; and flash (SPI), SDRAM, USB, and UART controllers. The advanced microcontroller bus architecture (AMBA) was used to implement the master–slave AHB protocols. The FPGA design’s overall block diagram is depicted in Figure 4. The FPGA design elements such as the maximum frequency; quantization; details regarding the CLB LUTs; CLB registers utilized; and DSPs, BRAM, etc., are shown in Table 2.

### 5.2. Flow of Data in FPGA Board

**Figure 5:**
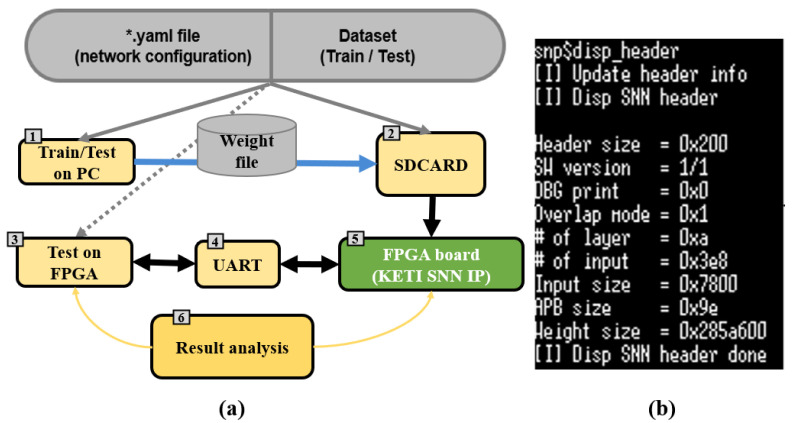
The data flow in the FPGA board. (**a**) Data pipeline for training and testing scenarios where * represent the name of the .yaml file and numbers represent the order of the tasks in a sequence; (**b**) FPGA SNN environment with header and deployment arguments.

**Figure 6:**
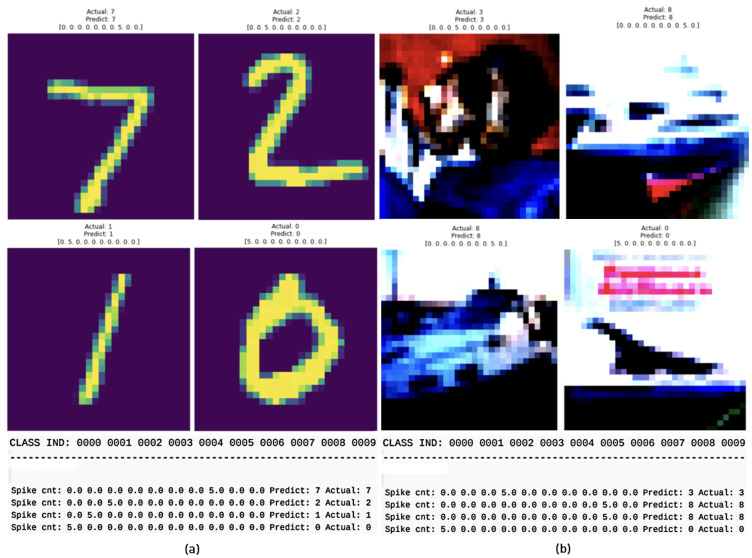
Spike-based data prediction on FPGA board. (**a**) Spike prediction on FPGA board (MNIST); (**b**) spike prediction on FPGA board (CIFAR10).

The training of the DCSNN was carried out on a PC with an NVIDIA GPU leveraging the processing power utilized for the backpropagation. The data flow in the FPGA board is depicted in Figure 5. Both the training and testing datasets were deployed for learning and inference purposes, respectively, on the PC to obtain initial estimates. In the next step, the trained model weight file (.bin) was transferred to the SD card and inserted into the FPGA board. The SD card created an environment inside the FPGA with arguments such as header, APB set, layer parameters, and inputs, which allowed us to run the SNN model. The data prediction process based on the underlying spiking mechanism on the FPGA is shown in Figure 6. The highest spike count in a specific category provided the prediction over the corresponding class. Additionally, a GUI demo video visualizing the inference data transfer from the FPGA to the PC via the UART can be found using the following link: https://github.com/INHACVLAB/DCSNN-on-FPGA/blob/main/SNN%20Object%20Classification%20(KITTI%20Dataset)%20on%20FPGA.mp4, accessed on 29 September 2022. The spiking activity that has the highest numerical value corresponds to the predicted class, and the resultant membrane potential values relate to the firing neuron activity during the prediction of the class.

### 5.3. DCSNN Architecture and Network Parameters

A significant number of papers have been published using the spike-timing-dependent plasticity (STDP) technique to deploy SNNs on an FPGA. However, the STDP does not conform to DCSNNs’ use of backpropagation. TSSL-BP is a well-designed backpropagation algorithm that supports significant open-source datasets. Therefore, TSSL-BP was evaluated and modified to match the FPGA deployment routines. On the other hand, the SGD-BP was chosen because we had conducted a substantial quantity of research utilizing this backpropagation technique, thus targeting better FPGA optimization. The TSSL-BP PC implementation and SGD-BP implementation codes can be found at https://github.com/INHACVLAB, accessed on 29 September 2022. The TSSL-BP-designed DCSNN architecture consisted of ten layers, which are depicted in Table 3 along with pertinent network characteristics. For the processing of a TSSL-BP coupled network, a total of 21,150,496 parameters are required.

The ten layers of the SGD-BP-designed DCSNN architecture are displayed in Table 4 along with pertinent network characteristics. A total of 5,322,592 parameters are required for the processing of an SGD-BP-coupled network. In TSSL-BP, the input image is directly fed into the convolution filters, where real-valued spikes are processed during future training, as described in [49]. In contrast, SGD-BP encodes the input image using Poisson distribution for the LIF neurons and simultaneously feeds the input image into the convolution and pooling layers. As stated in [47], the spike currents were calculated via a cumulative process incorporating LIF and convolutions, and the threshold was applied to the membrane potential.

## 6. Experiments and Results

### 6.1. Public and Private Datasets

The publicly available datasets included MNIST [61], CIFAR10 [62], and KITTI [63]. The MNIST dataset was considered to offer the classification scope of 10 classes from 0 to 9, and CIFAR10 contains classes such as airplane, automobile, bird, cat, deer, dog, frog, horse, ship, and truck. Similarly, the KITTI dataset consists of classes such as vehicle, cyclist, and pedestrian. Internally, cars, buses, and trucks were combined as vehicles for a generalized classification scenario with the privately-acquired INHA_ADAS dataset containing the same three classes of vehicle, cyclist, and pedestrian. The private datasets used in these experiments were designed for the sole purpose of classification tasks in the context of autonomous vehicle scenarios. The datasets such as INHA_ADAS and INHA_KLP were customized to assist the experiments related to assessing the classification performance of the DCSNNs. The INHA_ADAS dataset consisted of three classes, namely vehicle, cyclist, and pedestrian, which were maintained in correlation to the classes defined in the KITTI public dataset. Additionally, the INHA_KLP dataset was chosen and customized to test the inference capabilities of the DCSNNs for Korean license plates. A total of 50 classes were considered with the combination of numbers 0 to 9, just as in the case of the public MNIST set, and 40 Korean alphabet classes, which acted as the perfect scope to test the inference of the DCSNNs. The overall dataset specifications are stated in Table 5.

### 6.2. Performance Evaluations

**Figure 7:**
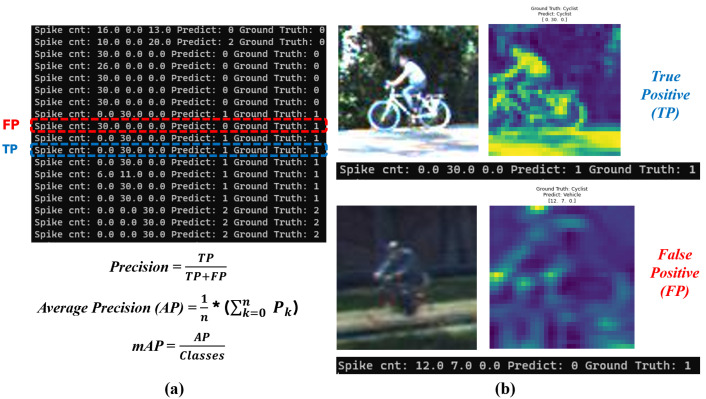
Spike-based mAP metric calculation. (**a**) Spike count and relevant mAP calculations; (**b**) true-positive and false-positive predictions.

The evaluations were carried out using mainly accuracy and processing time as fundamental performance criteria. The algorithms and networks were deployed on the FPGA for inference, and corresponding metrics related to the accuracy and processing time were collected for evaluation. The experiments were carried out on diverse datasets, and comparisons between the PC and FPGA with respect to TSSL-BL and SGD-BP were drawn. Usually, the mAP in traditional machine learning applications is calculated using a ground-truth bounding box. However, the mAP metric considered in this work was calculated according to the true-positive (TP) and false-positive (FP) precision values with respect to classes based on spike predictions. The mAP calculation is clearly illustrated with a use case in Figure 7, where the spike count led to the predicted class and thereby the mAP was calculated from the average precision. Moreover, the average power consumption (in Watts) was calculated on a CPU (Intel i7-12700) alongside FPGA Xilinx Kintex UltraScale (xcku115-flvf1924-2-i).

**Figure 8:**
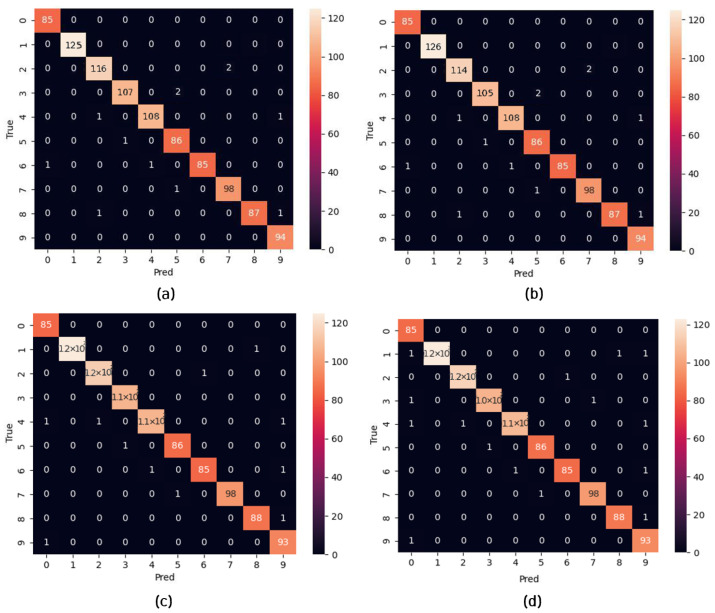
Confusion matrices. (**a**) TSSL-BP inference on the MNIST dataset (PC); (**b**) TSSL-BP inference on the MNIST dataset (FPGA); (**c**) SGD-BP inference on the MNIST dataset (PC); (**d**) SGD-BP inference on the MNIST dataset (FPGA).

**Figure 9:**
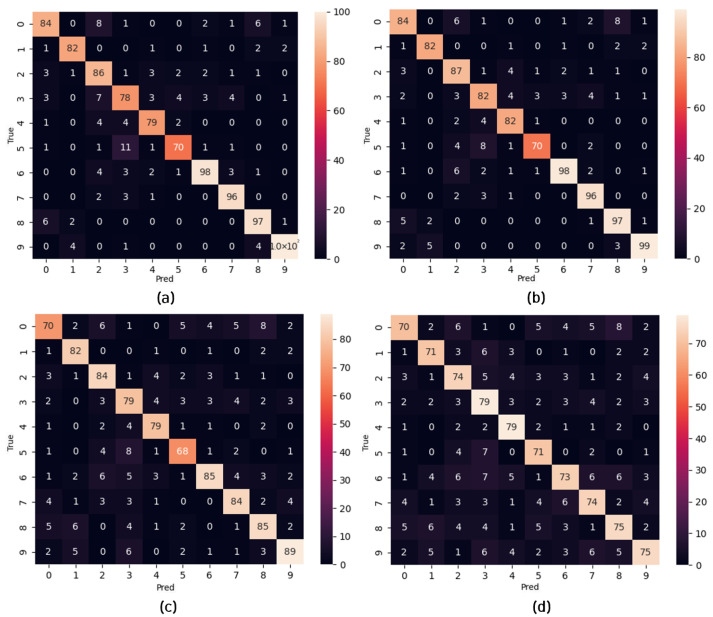
Confusion matrices. (**a**) TSSL-BP inference on the CIFAR10 dataset (PC); (**b**) TSSL-BP inference on the CIFAR10 dataset (FPGA); (**c**) SGD-BP inference on the CIFAR10 dataset (PC); (**d**) SGD-BP inference on the CIFAR10 dataset (FPGA).

**Figure 10:**
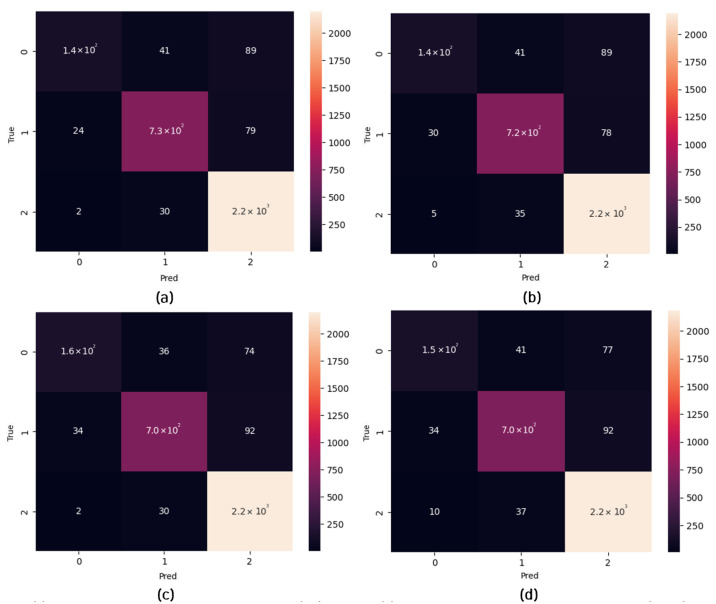
Confusion matrices. (**a**) TSSL-BP inference on the KITTI dataset (PC); (**b**) TSSL-BP inference on the KITTI dataset (FPGA); (**c**) SGD-BP inference on the KITTI dataset (PC); (**d**) SGD-BP inference on the KITTI dataset (FPGA).

Regarding the public dataset evaluations, Figure 8 shows the classification performance of the TSSL-BP- and SGD-BP-coupled DCSNNs on the MNIST dataset with 10 classes. Also, Table 6 presents the metrics corresponding to the samples inferred on the PC alongside the FPGA in terms of accuracy and processing time. Similarly, the confusion matrices in Figure 9 provide a glimpse of the classification performance of the TSSL-BP- and SGD-BP-coupled DCSNNs on the CIFAR10 dataset with 10 classes. Also, Table 7 presents the metrics corresponding to the samples inferred on the PC alongside the FPGA in terms of accuracy and processing time. Additionally, Figure 10 provides an overview of the classification performance of the TSSL-BP- and SGD-BP-coupled DCSNNs on the KITTI dataset with three classes. Also, Table 8 presents the metrics corresponding to the samples inferred on the PC alongside the FPGA in terms of accuracy and processing time. The latency of the DCSNN on the FPGA was calculated with respect to various datasets alongside the PC inference and was defined as the spiking FPGA inference latency with respect to the spiking PC inference. For instance, in Table 6, the FPGA latency with respect to MNIST for TSSL-BP is 49 times that of the PC.

**Figure 11:**
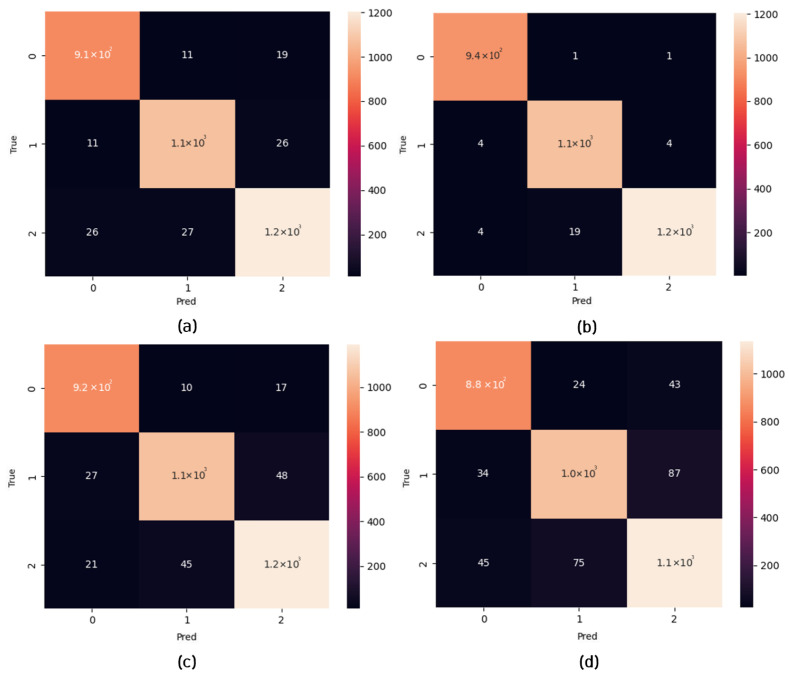
Confusion matrices. (**a**) TSSL-BP inference on the INHA_ADAS dataset (PC); (**b**) TSSL-BP inference on the INHA_ADAS dataset (FPGA); (**c**) SGD-BP inference on the INHA_ADAS dataset (PC); (**d**) SGD-BP inference on the INHA_ADAS dataset (FPGA).

**Figure 12:**
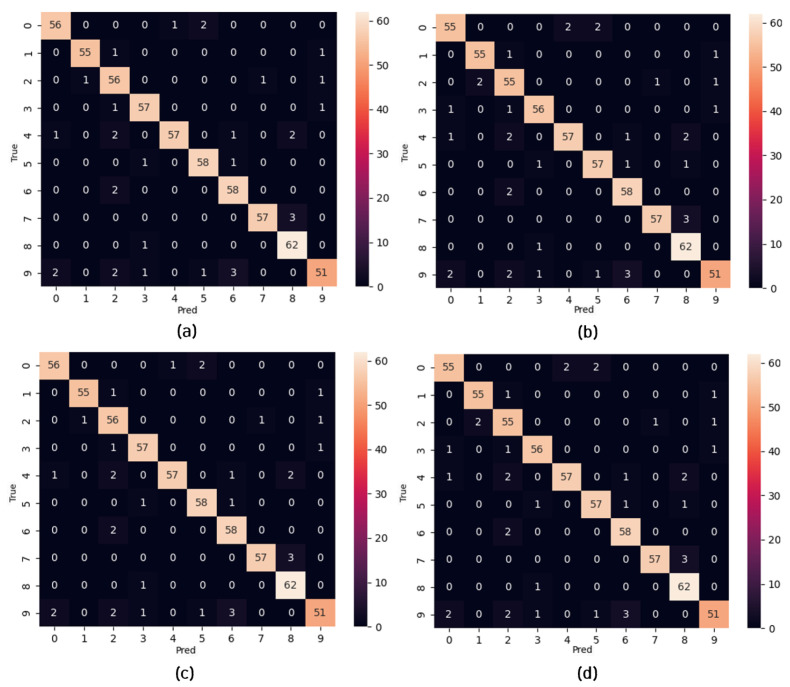
Confusion matrices. (**a**) TSSL-BP inference on the INHA_KLP dataset (PC); (**b**) TSSL-BP inference on the INHA_KLP dataset (FPGA); (**c**) SGD-BP inference on the INHA_KLP dataset (PC); (**d**) SGD-BP inference on the INHA_KLP dataset (FPGA).

Regarding the private dataset evaluations, the confusion matrices in Figure 11 show the classification performance of the TSSL-BP- and SGD-BP-coupled DCSNNs on the INHA_ADAS dataset with three classes. Also, Table 9 presents the metrics corresponding to the samples inferred on the PC alongside the FPGA in terms of accuracy and processing time. Figure 12 shows the classification performance of the TSSL-BP- and SGD-BP-coupled DCSNNs on the INHA_KLP dataset with 50 classes. Also, Table 10 presents the metrics corresponding to the samples inferred on the PC alongside the FPGA in terms of accuracy and processing time.

## 7. Comparative Study of BP Techniques (TSSL-BP vs. SGD-BP)

### 7.1. Classification Accuracy

**Figure 13:**
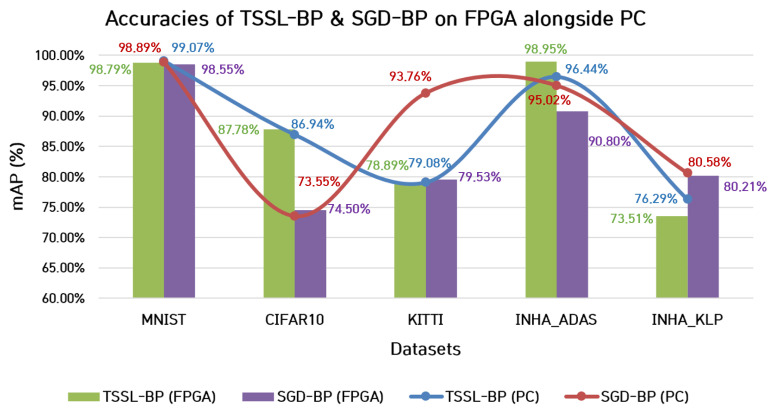
Comparison between classification accuracies (mAP%) of TSSL-BP and SGD-BP on FPGA alongside PC.

The classification accuracy of both backpropagation techniques is shown in Figure 13, where the green and purple column bar charts correspond to the performance of TSSL-BP and SGD-BP on the FPGA hardware, respectively. The classification accuracies of TSSL-BP on the datasets CIFAR10 and INHA_ADAS were higher than the classification accuracies of SGD-BP on the FPGA platform. However, the mAP metrics on the dataset INHA_KLP favored SGD-BP over TSSL-BP on the FPGA platform. Nevertheless, under the FPGA inference environment, the classification accuracies of both backpropagation techniques were similar on the datasets MNIST and KITTI.

#### 7.1.1. Processing Time

**Figure 14:**
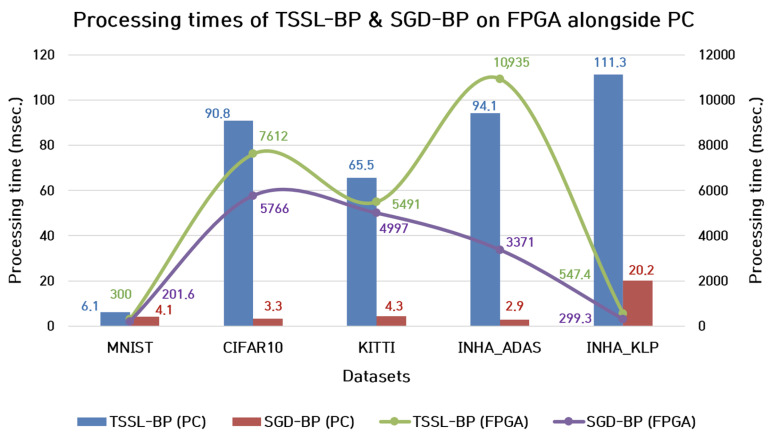
Comparison between processing times (msec) of TSSL-BP and SGD-BP on FPGA alongside PC.

Additionally, the inference processing time of both backpropagation techniques were estimated in the FPGA environment. The green and purple line plots in Figure 14 correspond to the inference processing times of TSSL-BP and SGD-BP on the FPGA hardware, respectively. The processing times of TSSL-BP on almost all the datasets were higher than the inference processing times of SGD-BP on the FPGA hardware. This suggested that the inference time taken for SGD-BP to classify a sample from all the diverse datasets was shorter than the inference time taken for TSSL-BP on the FPGA platform.

#### 7.1.2. Trade-Off between Accuracy and Processing Time

**Figure 15:**
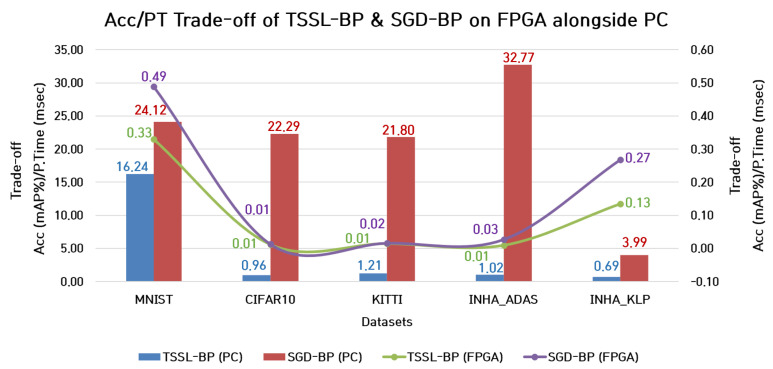
Comparison between trade-off metrics of TSSL-BP and SGD-BP on FPGA alongside PC.

The trade-off between the accuracy and processing time must be investigated thoroughly because of the ambiguity that might alter the choice of a proper backpropagation technique for coupling with DCSNNs to be deployed in classification tasks. For certain datasets with simple backgrounds and binary images as samples, the classification task can be considered less complex than in the case of complex backgrounds with clutter in color image samples. Also, the design elements such as the choice of hyperparameters and design options such as dropout rate and batch normalization can also influence the overall classification accuracy. However, the employment of low-cost FPGA platforms for the classification task converts the network design aspect into a complex set of restrictions. The hardware restrictions, such as the inability of the FPGA board to handle batch norms, dropout, and several other factors, are responsible for better accuracy. Therefore, instead of considering classification performance or processing time alone, it is appropriate to consider the trade-off between classification accuracy and processing time for a better choice of backpropagation technique. The trade-off metric can be estimated by the fraction term, with the numerator being the classification accuracy and the denominator being the processing time. To attain a better accuracy, the mAP is required to be as high as possible, and the processing time as low as possible. Accordingly, the trade-off is estimated by

(13) $$\psi =\frac{Acc(mAP\%)}{PT\left(\mathrm{msec}\right)},$$

where ψ is the trade-off metric, *Acc*(*mAP%*) refers to the classification accuracy (*mAP%*), and *PT* is the processing time in msec. This metric must be as high as possible, with the classification accuracy (*mAP%*) as the numerator and the processing time (msec) as the denominator. The comparisons between the trade-off metrics corresponding to TSSL-BP and SGD-BP on the FPGA and PC are shown in Figure 15. The blue and red column bar charts represent the trade-of metrics of TSSL-BP and SGD-BP in the PC environment, respectively. Similarly, the green and purple line plots represent the trade-off metrics of TSSL-BP and SGD-BP on the FPGA board, respectively.

### 7.2. Performance Analysis with Respect to Datasets on the FPGA Platform

The classification performance of both techniques varied with the datasets and other design aspects when deployed on the FPGA. A clear analysis of which technique should be preferred according to the classification accuracy on the FPGA is shown in Table 11. The table indicates the suitability of certain techniques when employed on the FPGA board in conjunction with specific datasets for better classification performance. Similarly, SGD-BP could be considered as a suitable candidate for coupling with DCSNNs deployed on FPGA platforms for classifying samples from KITTI, INHA_KLP, and MNIST. Both backpropagation techniques required considerable inference times to classify the samples on the FPGA board with respect to diverse datasets. These inference values are presented in Table 12, which clearly states that the processing time taken by SGD-BP to classify the samples from all five datasets on the FPGA board was far lower than that of TSSL-BP. Analogous to the processing time metric, the trade-off metric of SGD-BP seemed to contain better quantitative values compared to the TSSL-BP trade-off metric. These trade-off values are shown in Table 13, which clearly states that SGD-BP could be a suitable candidate for coupling with DCSNNs to achieve better performance on classification tasks in the context of FPGA environments for datasets such as MNIST, KITTI, INHA_ADAS, and INHA_KLP. In the tables below, the symbols + and − correspond to suitable and unsuitable BP candidates for the respective dataset.

### 7.3. Performance Analysis of the Current Study Alongside Other Works

Due to the current lack of open-source code for FPGA inference on certain datasets, two works [54,55] were chosen to evaluate the performance with respect to MNIST and a single work [55] with respect to CIFAR10. The relevant hardware specifications of these works are reported in Table 1. The evaluations were conducted in terms of quantization (bits), the number of parameters, accuracy (%), and throughput (frames per second). Table 14 and Table 15 show the corresponding results. However, with proper open-source algorithms, performance evaluations compared to other SNN works on FPGAs could be carried out with better precision in the future.

**Figure 16:**
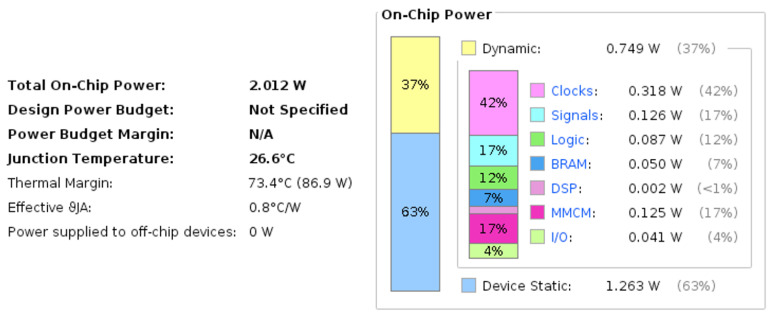
FPGA (xcku115-flvf1924-2-i) power utilization in dynamic mode (deploying DCSNNs).

In addition to the performance evaluations of contemporary studies, the power consumption aspect of the FPGA board was tested during this investigative study. The images in the public and private datasets were standard 32 × 32 pixel images. This size was maintained in all the experiments conducted in this study. The precision of 16-bit and the aforementioned image size was maintained while measuring the power consumption on the major computation devices used in this study. An Intel i7-12700 CPU and Xilinx Kintex UltraScale FPGA (xcku115-flvf1924-2-i) were used to run the DCSNNs with input images taken from all the datasets. As the input size was standardized on all the datasets, the power consumption was the same for all the datasets with respect to the computation device. In the comparative study, it was evident that the Xilinx Kintex UltraScale FPGA (xcku115-flvf1924-2-i) consumed much less power compared to the Intel i7-12700 CPU, and this could be quantified as being 18 times the power efficient, as shown in Table 16. To examine the on-chip power utilization percentage on the overall FPGA, the utilization metrics were acquired from the chip, and it appeared to be using 0.74 watts of dynamic power while deploying the DCSNN on the FPGA board, as shown in Figure 16.

## 8. Discussions, Limitations, and Future Work

This study focused on the deployment of DCSNNs on a low-cost FPGA board and reported the accuracy and processing time latency with respect to the hardware. The MNIST, CIFAR10, KITTI, INHA_ADAS, and INHA_KLP datasets were used to inform researchers and enterprises about the limitations and unique perspectives of this methodology. This experimental study had two primary objectives: (i) to determine the most effective backpropagation technique, TSSL-BP or SGD-BP, for deeper SNNs with convolution filters across multiple datasets; and (ii) to evaluate the feasibility of deploying DCSNNs trained using backpropagation techniques on low-power FPGAs for inference, taking into account potential configuration adjustments and power consumption. The inference performed on the FPGA necessitated the customization of networks with respect to constraints such as batch normalization. The network porting (.yaml) file required to operate the DCSNN on an FPGA can be accessed via the following link: https://github.com/INHACVLAB/DCSNN-on-FPGA/tree/main/networks, accessed on 29 September 2022. When using low-cost FPGA devices to implement DCSNNs for classification tasks, the trade-off between accuracy and the processing time is crucial. The processing time attribute varied depending on the dataset and has been depicted as a latency parameter in various tables, such as Table 6, Table 7, Table 8, Table 9 and Table 10. The processing duration of the model deployed on an FPGA was at least 50 times shorter than the model deployed on a PC. Due to the dearth of open-source FPGA-related working codes, the performance analysis was limited to a small number of recent works. The outcomes depicted in Table 14 and Table 15 served as the performance analysis of the current study compared to other works with respect to the MNIST and CIFAR10 datasets on an FPGA board. As shown in Table 16, the power efficiency of the Xilinx Kintex UltraScale FPGA (xcku115-flvf1924-2-i) was 18 times that of the Intel CPU (i7-12700) for all datasets with an input image size of 32 × 32 and 16-bit precision. This is an essential observation for the investigation of the performance of custom FPGAs using DCSNN models.

The private datasets, such as INHA_ADAS and INHA_KLP, aided in the exploration of BP techniques in general on the PC and later on the FPGA, which provided insights into the feasibility of employing BP techniques in future experiments. In addition to the topics discussed, there were a few limitations associated with the current study, including:

1. The current work was limited to testing DCSNNs on a single FPGA model, Xilinx Kintex UltraScale. Due to the lack of open-source code, the performance analysis conducted in the study was unable to fully address the pros and cons of the model in comparison to contemporary works carried out on other FPGA models. In the future, this issue could be effectively resolved by contemplating multiple models of FPGA boards with similar on-chip SNN deployment design elements and evaluating various DCSNNs with respect to various datasets.
2. Experiments must be conducted to ensure that the surrogate gradient descent backpropagation technique is well-tuned to enhance classification accuracy on several ADAS-based private datasets while preserving the shallower network design layers.
3. Deeper networks (DCSNNs) are currently considered for massive datasets using TSSL-BP and SGD-BP. However, the network design could be expanded to shallow layered networks using the customized parametric surrogate gradient descent backpropagation technique (CPSGD-BP) for greater data size flexibility without compromising performance.

## 9. Conclusions

Deep convolutional neural networks utilizing spike-based backpropagation techniques such as TSSL-BP and SGD-BP were successfully implemented and deployed on the Xilinx Kintex UltraScale FPGA platform. The efficacy of the DCSNN in terms of classification accuracy and processing time was evaluated using a variety of metrics on public and private datasets. Using both a PC and FPGA, comparative deployment studies of spike-based backpropagation-coupled DCSNNs on various datasets were conducted, and the results were documented in terms of classification accuracy, processing time, and the trade-off metric. Similarly, a performance analysis of the current study was conducted alongside other works on the MNIST and CIFAR10 datasets. For the purpose of evaluating BP-trained DCSNNs deployed on FPGAs in relation to public and private datasets, all conceivable evaluation methods were investigated. Finally, the potential future directions that could aid researchers attempting to develop DSCNNs for FPGAs, with or without BP techniques, were discussed. Also, the current work validated performance using metrics focusing on accuracy, processing time, and the trade-off between them; however, future work will need to employ multiple hardware metrics on diverse datasets. This investigation into deploying DCSNNs on a low-cost FPGA board and determining the accuracy and processing time latency with respect to the MNIST, CIFAR10, KITTI, INHA_ADAS, and INHA_KLP datasets can inform researchers and businesses about the limitations and unique perspectives of this approach. In the future, there is a need for diverse optimization methods to reduce latency by sustaining the accuracy and low-power characteristics of FPGAs to benefit the medium-scale intelligent vehicle industry.

## Figures and Tables

**Table 1:** Literature review of works deploying SNNs and hybrid networks on FPGA platforms alongside this study.

Network Type	Hybrid [51]	Hybrid [52]	Hybrid [53]	Hybrid [54]	SNN [57]	SNN [55]	Hybrid [56]	Hybrid [49]	Hybrid (This Work)
FPGA Model	Xilinx Artix-7	XCVU440	Xilinx Virtex-6 ML605	Xilinx Zynq UltraScale +XCZU7EV	Xilinx ZCU104	Xilinx VC707 Xilinx ZCU102 Xilinx VCU118	Xilinx Zynq Ultrascale+	Xilinx Kintex UltraScale FPGA	Xilinx Kintex UltraScale FPGA xcku115-flvf1924-2-i
Datasets	Synthetic	MNIST SVHN CIFAR10	DARPA CIFAR10	MNIST CIFAR10 ImageNet	MNIST CIFAR10	MNIST SVHN CIFAR10	MNIST	MNIST CIFAR10	MNIST CIFAR10 KITTI INHA_ADAS INHA_KLP
Encoding Scheme	Real-value spike	Real-value spike	Real-value spike	m-TTFS	Real-value spike	Real-value spike	Rate encoding	Real-value spike	Real-value and Poisson spike
Neuron Model	IF	IF	IF	LIF	IF	Izhikevich	LIF	LIF	LIF
Convolution Filter Optimization	Time division multiplexing (TDM)	None	None	None	None	None	Unrolling	None	None

**Table 2:** FPGA design elements.

Design Aspect	Value
Device model	Xilinx Kintex UltraScale FPGA (xcku115-flvf1924-2-i)
Maximum frequency	80 Mhz
Quantization	16-bit fixed point
Synchronization	Clock-based synchronization
CLB LUTs	196,043
CLB Registers	172,011
CLB	32,102
DSP	415
BRAM	112 KB
SDRAM	256 MB
Spike timing per fixed window	12.5 ms
Input encoding scheme	Real-value spike encoding
Average latency to first spike	303 ms

**Table 3:** DCSNN architecture coupled with TSSL-BP and network parameters.

Input	Output	Layer	[Kernel, Stride]	Parameters
(32, 32, 3)	(32, 32, 96)	conv3-96	[3 × 3, 1]	2592
(32, 32, 96)	(16, 16, 256)	conv3-256	[3 × 3, 1]	221,440
(32, 32, 256)	(32, 32, 256)	pooling	[2 × 2, 3]	0
(16, 16, 256)	(8, 8, 384)	conv3-384	[3 × 3, 1]	885,120
(16, 16, 384)	(16, 16, 384)	pooling	[2 × 2, 3]	0
(8, 8, 384)	(8, 8, 384)	conv3-384	[3 × 3, 1]	1,327,488
(8, 8, 384)	(8, 8, 16,384)	conv3-256	[3 × 3, 1]	884,992
(1, 1, 16,384)	(1, 1, 1024)	fc	[1 × 1, 0]	16,777,216
(1, 1, 1024)	(1, 1, 1024)	fc	[1 × 1, 0]	1,048,576
(1, 1, 1024)	(1, 1, 3)	fc	[1 × 1, 0]	3072

**Table 4:** DCSNN architecture coupled with SGD-BP and network parameters.

Input	Output	Layer	[Kernel, Stride]	Parameters
(32, 32, 3)	(32, 32, 32)	conv3-32	[3 × 3, 1]	864
(32, 32, 32)	(32, 32, 32)	LIF-neuron	none	0
(32, 32, 32)	(32, 32, 64)	conv3-64	[3 × 3, 1]	18,432
(32, 32, 64)	(32, 32, 64)	LIF-neuron	none	0
(32, 32, 64)	(16, 16, 64)	Avg.pooling	[2 × 2, 2]	0
(16, 16, 64)	(16, 16, 128)	conv3-128	[3 × 3, 1]	73,728
(16, 16, 128)	(16, 16, 128)	LIF-neuron	none	0
(16, 16, 128)	(16, 16, 128)	conv3-128	[3 × 3, 1]	147,456
(16, 16, 128)	(16, 16, 128)	LIF-neuron	none	0
(16, 16, 128)	(8, 8, 128)	Avg.pooling	[2 × 2, 2]	0
(8, 8, 128)	(8, 8, 256)	conv3-256	[3 × 3, 1]	294,912
(8, 8, 256)	(8, 8, 256)	LIF-neuron	none	0
(8, 8, 256)	(8, 8, 256)	conv3-256	[3 × 3, 1]	589,824
(8, 8, 256)	(8, 8, 256)	LIF-neuron	none	0
(8, 8, 256)	(4, 4, 256)	Avg.pooling	[2 × 2, 2]	0
(4, 4, 256)	(1, 1, 4096)	flatten	none	0
(1, 1, 4096)	(1, 1, 1024)	fc	[1 × 1, 0]	4,194,304
(1, 1, 1024)	(1, 1, 1024)	LIF-neuron	none	0
(1, 1, 1024)	(1, 1, 1024)	dropout	none	0
(1, 1, 1024)	(1, 1, 3)	fc	[1 × 1, 0]	3072

**Table 5:** Datasets used in the current work.

Dataset	Category	Classes	No. of Samples
MNIST	Public	10	70,000
CIFAR10	Public	10	60,000
KITTI	Public	3	48,100
INHA_ADAS	Private	3	30,722
INHA_KLP	Private	50	48,100

**Table 6:** Performance of TSSL-BP (rows 1 and 2) and SGD-BP (rows 3 and 4) on the MNIST dataset.

Platform	Mean (%)	Best (%)	mAP (%)	Processing Time (ms)	Power Consumption (W)
CPU (Intel i7-12700)	99.10	99.30	99.07	6.1	13.6
FPGA (xcku115-flvf1924-2-i)	98.50	98.80	98.79	300	0.74
CPU (Intel i7-12700)	99.1	99.15	98.89	4.1	12.91
FPGA (xcku115-flvf1924-2-i)	98.5	98.80	98.55	201.6	0.74
Latency of TSSL-BP on FPGA with respect to MNIST dataset				49×	
Latency of SGD-BP on FPGA with respect to MNIST dataset				50×	
Average power efficiency of TSSL-BP on FPGA with respect to MNIST dataset					18×
Average power efficiency of SGD-BP on FPGA with respect to MNIST dataset					18×

**Table 7:** Performance of TSSL-BP (rows 1 and 2) and SGD-BP (rows 3 and 4) on the CIFAR10 dataset.

Platform	Mean (%)	Best (%)	mAP (%)	Processing Time (ms)	Power Consumption (W)
CPU (Intel i7-12700)	86.90	87.00	86.94	90.8	13.6
FPGA (xcku115-flvf1924-2-i)	87.00	87.80	87.78	7612	0.74
CPU (Intel i7-12700)	73.72	74.82	73.55	3.3	12.91
FPGA (xcku115-flvf1924-2-i)	74.51	75.02	74.50	5766	0.74
Latency of TSSL-BP on FPGA with respect to CIFAR10 dataset				83×	
Latency of SGD-BP on FPGA with respect to CIFAR10 dataset				1747×	
Average power efficiency of TSSL-BP on FPGA with respect to CIFAR10 dataset					18×
Average power efficiency of SGD-BP on FPGA with respect to CIFAR10 dataset					18×

**Table 8:** Performance of TSSL-BP (rows 1 and 2) and SGD-BP (rows 3 and 4) on the KITTI dataset.

Platform	Mean (%)	Best (%)	mAP (%)	Processing Time (ms)	Power Consumption (W)
CPU (Intel i7-12700)	86.17	86.52	79.2	65.5	13.6
FPGA (xcku115-flvf1924-2-i)	85.72	86.80	78.89	5491	0.74
CPU (Intel i7-12700)	97.31	97.45	80.58	4.3	12.91
FPGA (xcku115-flvf1924-2-i)	79.4	79.82	79.53	4997	0.74
Latency of TSSL-BP on FPGA with respect to KITTI dataset				83×	
Latency of SGD-BP on FPGA with respect to KITTI dataset				1162×	
Average power efficiency of TSSL-BP on FPGA with respect to KITTI dataset					18×
Average power efficiency of SGD-BP on FPGA with respect to KITTI dataset					18×

**Table 9:** Performance of TSSL-BP (rows 1 and 2) and SGD-BP (rows 3 and 4) on the INHA_ADAS dataset.

Platform	Mean (%)	Best (%)	mAP (%)	Processing Time (ms)	Power Consumption (W)
CPU (Intel i7-12700)	97.44	97.89	96.5	94.1	13.6
FPGA (xcku115-flvf1924-2-i)	97.8	98.3	98.95	10,935	0.74
CPU (Intel i7-12700)	99.18	99.15	95.02	2.9	12.91
FPGA (xcku115-flvf1924-2-i)	95.4	96.01	90.8	3771	0.74
Latency of TSSL-BP on FPGA with respect to INHA_ADAS dataset				115×	
Latency of SGD-BP on FPGA with respect to INHA_ADAS dataset				1300×	
Average power efficiency of TSSL-BP on FPGA with respect to INHA_ADAS dataset					18×
Average power efficiency of SGD-BP on FPGA with respect to INHA_ADAS dataset					18×

**Table 10:** Performance of TSSL-BP (rows 1 and 2) and SGD-BP (rows 3 and 4) on the INHA_KLP dataset.

Platform	Mean (%)	Best (%)	mAP (%)	Processing Time (ms)	Power Consumption (W)
CPU (Intel i7-12700)	88.21	88.27	74.06	111.3	13.6
FPGA (xcku115-flvf1924-2-i)	87.00	88.27	73.51	547.4	0.74
CPU (Intel i7-12700)	98.24	98.46	80.58	20.2	12.91
FPGA (xcku115-flvf1924-2-i)	80.51	81.33	80.21	299.3	0.74
Latency of TSSL-BP on FPGA with respect to INHA_KLP dataset				5×	
Latency of SGD-BP on FPGA with respect to INHA_KLP dataset				14×	
Average power efficiency of TSSL-BP on FPGA with respect to INHA_KLP dataset					18×
Average power efficiency of SGD-BP on FPGA with respect to INHA_KLP dataset					18×

**Table 11:** Suitability of BP techniques according to their classification performance on the FPGA platform with respect to various datasets.

Technique	MNIST	CIFAR10	KITTI	INHA_ADAS	INHA_KLP
TSSL-BP	+	+	−	+	−
SGD-BP	+	−	+	−	+

**Table 12:** Suitability of BP techniques according to their inference time on the FPGA platform with respect to various datasets.

Technique	MNIST	CIFAR10	KITTI	INHA_ADAS	INHA_KLP
TSSL-BP	−	−	+	−	−
SGD-BP	+	+	+	+	+

**Table 13:** Suitability of BP techniques according to their trade-off metric on the FPGA platform with respect to various datasets.

Technique	MNIST	CIFAR10	KITTI	INHA_ADAS	INHA_KLP
TSSL-BP	−	+	−	−	−
SGD-BP	+	+	+	+	+

**Table 14:** Performance analysis of the current study alongside other works with respect to MNIST dataset.

Category	Sommer et al. [54]	Aung et al. [55]	TSSL-BP [49]	SGD-BP [47]
Model	Xilinx Zynq UltraScale +XCZU7EV	Xilinx VC707	Xilinx Kintex UltraScale	Xilinx Kintex UltraScale
Quantization	16 bits	8 bits	16 bits	16 bits
Weight	None	1.17M	21.1M	1.38M
Accuracy	98.2%	98.1%	98.7%	97.8%
Throughput	21 FPS	33 FPS	3.3 FPS	3.5 FPS

**Table 15:** Performance analysis of the current study alongside other works with respect to CIFAR10 dataset.

Category	Aung et al. [55]	TSSL-BP [49]	SGD-BP [47]
Model	Xilinx VCU118	Xilinx Kintex UltraScale	Xilinx Kintex UltraScale
Quantization	8 bits	16 bits	16 bits
Weight	12M	21.1M	1.38M
Accuracy	81.8%	87.7%	78.8%
Throughput	4.04 FPS	0.13 FPS	0.17 FPS

**Table 16:** Power efficiency of FPGA (xcku115-flvf1924-2-i) compared to CPU (Intel i7-12700).

Computation Device (Type and Model)	Precision (Bits)	Input Image Size (Pixel)	Average Power Consumption (W)	
			TSSL-BP	SGD-BP
CPU (Intel i7-12700)	16 (same for all datasets)	32 × 32 (same for all datasets)	13.6	12.91
FPGA			0.74	0.74
(xcku115-flvf1924-2-i)				
The average power efficiency of FPGA was 18×			that of CPU across all the datasets	

## Data Availability

The data presented in this study are available on request from the corresponding author. The data are not publicly available due to ongoing validations and continuous improvements.

## Abbreviations

The following abbreviations are used in this manuscript:

DCSNN	Deep convolutional spiking neural network
TSSL-BP	Temporal spike sequence learning backpropagation
SGD-BP	Surrogate gradient descent via backpropagation
FPGA	Field-programmable gate array
MNIST	MNIST digit classification dataset
CIFAR10	CIFAR object classification dataset with 10 object categories
INHA_ADAS	Advanced driver assistance systems vehicle classification dataset collected by INHA University
INHA_KLP	Korean license plate alphabet classification dataset collected by INHA University
LIF neuron	Leaky integrate firing neuron
IF neuron	Integrate firing neuron
STDP	Spike-time-dependent plasticity
UART	Universal asynchronous receiver–transmitter
AMBA	Advanced microcontroller bus architecture
CLB	Configurable logic block
*v*	Presynaptic neuron
*u*	Postsynaptic neuron
$X_{v}\left(t\right)$	Input spike train
$f_{v}^{\left(t\right)}$	Firing time of presynaptic neuron
$J_{v}\left(t\right)$	Postsynaptic current
$P_{u}\left(t\right)$	Membrane potential voltage
$R_{o}$	Leaky resistance of the LIF neuron
$\tau_{p}$	Membrane potential time constant
$\tau_{s}$	Synaptic time constant
$Q_{uv}$	Weight of the synaptic connection
$r_{u}\left(t\right)$	Reset mechanism in the spiking activity
α(·)	Response mechanism kernel
β(·)	Reset mechanism kernel
$V_{Eq}$	Firing equilibrium
H(·)	Step function
$D_{sp}$	Distance between desired spikes
$S_{sp}$	Distance between produced (actual) spikes
$D_{sp}{|}_{t}$	Firing events for desired spikes
$S_{sp}{|}_{t}$	Firing events for produced (actual) spikes
$N_{t}$	Total time steps
$L_{temp.sp}$	Temporal spike loss function
$\xi_{TSSL}\left[t\right]$	TSSL error at time *t*
▵(·)	Van Rossum distance function
$\xi_{SGD}\left[t\right]$	SGD error at time *t*
$Z_{gt}$	Ground-truth classification labels
*Z*	Actual output of the network
χ	Hyperparameter
*c*	Gradient thickness
